# *In Vitro* Evaluation of Different Solvents for Retrieval of Mineral Trioxide Aggregate and Calcium-Enriched Mixture

**DOI:** 10.7508/iej.2016.03.015

**Published:** 2016-05-01

**Authors:** Nooshin Sadat Shojaee, Alireza Adl, Fereshte Sobhnamayan, Azam Khademi, Mina Hamedi

**Affiliations:** a *Department of Endodontics, Dental School, Shiraz University of Medical Sciences, Shiraz, Iran; *; b * Department of Endodontics, Biomaterials Research Center, Dental School, Shiraz University of Medical Sciences, Shiraz, Iran; *; c * Student, Dental School, Shiraz University of Medical Sciences, Shiraz, Iran*

**Keywords:** Calcium-Enriched Mixture, Microhardness, Mineral Trioxide Aggregate, Retrieval

## Abstract

**Introduction:**

The purpose of this study was to evaluate the effect of different solvents; carbonic acid (H_2_CO_3_), hydrochloric acid (HCl), chlorhexidine (CHX) and sodium hypochlorite (NaOCl) on the surface hardness of mineral trioxide aggregate (MTA) and calcium-enriched mixture (CEM) cement.

**Methods and Materials:**

Plexiglass molds were prepared and filled with Angelus MTA or CEM cement and then exposed to 2% carbonic acid, 37% hydrochloric acid, 2% chlorhexidine, 5.25% sodium hypochlorite and normal saline at intervals of 1 and 21 days, respectively (*n*=4). Surface microhardness of all specimens was analyzed by a universal testing machine and an electron microscope for some selected samples. Data were analyzed using the three-way ANOVA. Subgroup analysis was performed by Student’s t-test, One-way ANOVA and Tukey’s tests. The level of significance was set at 0.05.

**Results:**

On the first day, all solvents and on 21^st^ day HCl, and H_2_CO_3 _were more effective in reducing the microhardness of MTA compared to CEM cement (*P*<0.05).

**Conclusion:**

The two experimental cements were differently affected by the solvents at specific time intervals. The solvents were more effective on MTA.

## Introduction

The aim of root end filling is to seal the contents of the root canal system in order to prevent the egress of microorganisms or their byproducts into the periradicular tissues [1]. An ideal root-end filling material should be biocompatible, antibacterial, nontoxic and radiopaque and it should not be resorbable or soluble in the oral environment. Mineral trioxide aggregate (MTA) is one of the most popular root end filling materials [2]. This cement has a wide variety of applications, which can be attributed to its bioactivity [3], biocompatibility [4, 5] and radiopacity [6], good sealing ability [7, 8] and antibacterial efficiency [9].

Initial root canal therapy is a predictable procedure with a high degree of success, but sometimes failures can occur after treatment. Therefore; retrievability of the root-end filling is an important concern [10]. Moreover, it has been shown that MTA might lose its sealing ability and effective barrier thickness due to increasing solubility in the long term [11-13]. In this situations the clinician should refresh or even exchange previously applied MTA with new mixed cement in order to reestablish the sealing ability of this material. As MTA sets into a hard mass, its retrievability is very hard, and can pose significant procedural problems during retreatment [14]. It has been shown that rotary and ultrasonic instruments are not efficient in the complete removal of MTA from the root canal [15]. Recently, some studies have evaluated the effect of different solvents on the removal of MTA and showed that some acids and solvents can be successfully used for removal of MTA [4, 16, 17]. 

Calcium-enriched mixture (CEM) cement is another root end filling material with clinical uses similar to those of MTA. This material has good handling characteristics, sets in an aqueous environment and forms an effective seal at the root end [18]. Like MTA, CEM cement turns to a hard mass after setting, with a microhardness similar to MTA [19].

In one study exposure to butyric acid, to simulate infectious condition, reduced the surface microhardness of CEM cement [20]. Therefore, like MTA, CEM cement might interact with acids or chemicals and show disintegration. The aim of this study was to evaluate the dissolving ability of hydrochloric acid (HCl), carbonic acid (H_2_CO_3_), chlorhexidine gluconate (CHX), sodium hypochlorite solution (NaOCl) and saline on set 1- and 21-day CEM and MTA samples.

## Materials and Methods

The study was approved by the Vice Chancellor of Research in Shiraz University of Medical Sciences (Grant No: 8370). A total of 20 custom-made plexiglass molds were used in this experiment. According to ASTM E384 Standard for microhardness tests, each mold had four holes with an internal diameter of 4±0.1 mm and height of 6±0.1 mm (80 samples).

Angelus MTA (Angelus, Londrina, Paraná, Brazil) and CEM cement (BioniqueDent, Tehran, Iran) were mixed according to their manufacturers’ instructions. The molds were divided into two groups and filled with the mixed cements (*n*=40). To produce fully saturated humidity, moist cotton pellets were kept on the top of the condensed cements. The molds were then stored in a container and kept in an incubator at 37^°^C. The moist cotton pellets were changed every 3 days. The specimens of each group were divided to two subgroups and tested for hardness after 1 and 21 days of setting using Vickers microhardness testing machine (Bareiss Prufgeratebau GmbH, Oberdischingen, Germany).

All specimens were examined with a light microscope under 40× magnification. Samples with any defect or crack were excluded from the study. After polishing the samples, surface microhardness test was performed by using a Vickers Tester. A diamond indenter with a 50-g load and a dwell time of 10 sec was used. Three indentations were created on the polished surface of each sample at separate locations in accordance with ASTM E384 standard for Vickers microhardness test. A pilot test showed that this load creates a reliable indent in the specimens. The Vickers microhardness number (VHN) was calculated using the following formula: VHN=¼ 1:854×*L*⁄*d*^2^, where *L* is the applied load (kg) and *d* is the mean indentation diagonal length (mm).

These specimens were then randomly divided into five groups (*n*=4) and exposed to the experimental solvents: Group1: 37% HCl (pH=1.8) (Merck, Darmstadt, Germany); Group 2: 2% H_2_CO_3_ (pH=5.45); Group 3: 2% CHX (Merck, Darmstadt, Germany); Group 4: 5.25% NaOCl solution (Creamed, Poland); and Group 5 (control): normal saline (Darupakhsh, Tehran, Iran). The preparation of H_2_CO_3_ and HCl at the required concentrations was conducted in the Department of Pharmacology of Shiraz University of Medical Sciences.

The samples were continuously exposed to different solvents (one drop per min) for 15 min. Samples were then rinsed by distilled water for 1 min, dried and tested for microhardness. Data were collected and the mean microhardness values before and after exposure to the solvents for all the groups were calculated. Data were analyzed using the three-way ANOVA. As the three-way and all two-way interactions were significant, subgroup analysis was performed using the Student’s t-test for comparing the materials and effect of time and One-way ANOVA/Tukey’s post hoc test for comparing the solutions. The level of significance was set at 0.05.

***Scanning electron microscopy (SEM)***

Because of the large number of experimental groups, four specimens from experimental groups (MTA and CEM samples affected by NaOCl on day 1 and set MTA and CEM samples after 21 days, affected by HCl) were randomly selected for microstructural morphological evaluation under the scanning electron microscopy (SEM).

After the dislodgment of MTA and CEM cement, samples were irrigated with 10 mL of distilled water and prepared for evaluation by SEM. To analyze the internal microstructure, the specimens were vertically grooved on both sides with a disposable surgical scalpel blade to initiate a crack and then split longitudinally with a chisel. One-half of each sample was randomly selected, placed in 2% glutaraldehyde for 24 h, rinsed three times with sodium cacodylate buffer solution (0.1 M, pH=7.2), and then dehydrated with ethyl alcohol (30-100%). Finally, the specimens were placed in a desiccator for 24 h and mounted on a metallic stub. Then, the surfaces were coated with gold and SEM micrographs were taken under 250× magnification (Leo. 440i; Oxford Microscopy, Oxford, UK).

## Results

The mean VHN of samples before and after exposure to the solvents was calculated. For MTA samples the mean VHN before exposure were 35.81±1.87 and 48.84±5.12 on day 1 and 21, respectively. For CEM samples, mean VHN before exposure on day 1 and 21 were 23.48±1.31and 43.92±3.71, respectively. The mean changes in VHNs of specimens after exposure to the solvents in 1 and 21 days are summarized in Tables 1 and 2.

On day 1, all solvent were more effective on MTA than on CEM cement. In this time period and for MTA, all solvents significantly decreased the VHN values compared to saline (*P*<0.001). However, for CEM cement, only HCl and NaOCl caused a significant decrease in microhardness compared to other solvents (*P*<0.001).

On day 21, HCl and H_2_CO_3_ were more effective on MTA than CEM cement (*P*<0.001). In this time period, HCl and H_2_CO_3_ caused a significant reduction in the microhardness of MTA (*P*<0.001), while none of the solvents were effective on CEM cement (*P*>0.05).

***SEM*** ***analysis***

**Figure1:**
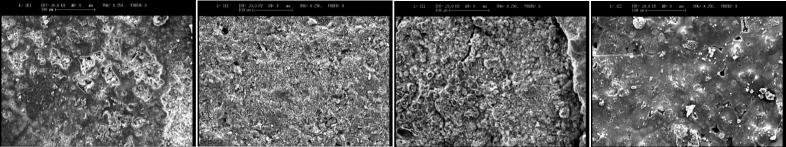
Scanning electron microscopy image of specimens of *A)* MTA affected by NaOCl after 1 day; and *B)* CEM affected by NaOCl after 1 day; *C)* MTA affected by HCl after 21 days and *D)* CEM affected by HCl after 21 days. Diffuse depressions and porosities can be seen (magnification 250

SEM micrographs of MTA and CEM samples affected by NaOCl on day 1 are shown in Figures 1A and 1B. Coarse crystalline structure was seen in both cements, but MTA samples affected by NaOCl seemed to have more porosity than CEM cement samples. Figures 1C and 1D show the SEM images of MTA and CEM specimens set after 21 days, affected by HCl. Evaluation of CEM micrographs showed more homogenous material with some channels and porosities that were probably produced by HCl. MTA specimens exposed to HCl had extensive diffuse micro-channels in a distinctive crystalline structure. 

## Discussion

In the present study, dissolution of MTA and CEM cement was assessed by reduction in their microhardness after exposure to various chemicals as solvents. Microhardness testing is based on evaluating the resistance of materials to deformation [21]. Therefore, it can be presumed that by decreasing the microhardness of cements they could be removed more easily. 

In this study the effects of two acids, two endodontic irrigants and normal saline, as control, were evaluated on the surface microhardness of MTA and CEM cement. H_2_CO_3_ is a weak acid with a pH of 5.48 and is a component of blood [22]. HCl is a well-known acid used in the industry for removal of cements and can be effectively used by passing it in contact with cement [23]. NaOCl and CHX are used as the most common irrigants in endodontics [24].

According to the results on day 1, all test materials (except for saline) caused a significant reduction in surface microhardness of MTA, while only HCl and NaOCl reduced the microhardness of CEM cement. It means that one day after application, when MTA and CEM are partially set, 15-min exposure to the mentioned chemicals facilitate removal of these cements

It should be noticed that in day 1, all used chemicals were more effective on MTA compared to CEM cement. Thus it can be concluded that when chemical solvents are used, retrieval of partially set MTA is more facilitated compared to CEM cement. 

On day 21, none of the solvents reduced the microhardness of CEM cement; however HCl and H_2_CO_3_ caused significant reductions in the microhardness of MTA. Therefore, none of chemical used in this study are useful as an adjunct to dissolve completely set CEM cement, while H_2_CO_3 _and HCl can be effectively used to dissolve completely set MTA.

The results of the present study also showed that CHX and NaOCl were able to reduce the microhardness of MTA only in the 1-day-set samples. These results are consistent with those of Butt *et al.* [17] who reported that 2% CHX and 5.25% NaOCl decreased the microhardness of partially set MTA. Nandidi *et al.* [22] also showed that 2% CHX reduced the surface hardness of set MTA significantly on day 1 only. Thus, usage of CHX and NaOCl as a root canal irrigants where using MTA is due during endodontic procedure, should be avoided for 24 h, unless for retrieval of the cement. On the other hand, in CEM cement groups, CHX caused no reduction and NaOCl reduced the microhardness only on day 1. Therefore using CHX as a root canal irrigant whenever CEM is being used, is safe. 

**Table 1.** Intergroup comparison of the reduction in mean (SD) of Vickers hardness of samples in the presence of different solvents after 1 day [different superscript uppercase letters (rows) or lowercase letters (columns) are significantly different (*P*<0.05

**Medium**	**MTA **	**CEM cement**
**HCl**	-14.69 (20794) ^aA^	-7.377 (1.756) ^aB^
**H**_2_ **CO**_3_	-8.54 (6.18) ^aA^	1.605 (0.990) ^bB^
**CHX**	-12.280 (2.919) ^aA^	0.275 (1.306) ^bB^
**NaOCl**	-10.172 (0.866) ^aA^	-7.475 (0.340) ^aB^
**Normal** ** saline**	-0.205 (1.162) ^bA^	3.387 (2.051) ^bB^

**Table 2:** Intergroup comparison of the reduction in mean (SD) of Vickers hardness of samples in the presence of different solvents after 21 days [different superscript uppercase letters (rows) or lowercase letters (columns) are significantly different (*P*<0.05)]

**Medium**	**MTA **	**CEM cement**
**HCl**	-26.835 (1.808) ^aA^	-2.912 (1.347) ^aB^
**H**_2_ **CO**_3_	-17.082 (0.249) ^bA^	-5.025 (3.640) ^aB^
**CHX**	3.345 (3.271) ^cA^	-1.812 (4.668) ^aA^
**NaOCl**	0.127 (1.246) ^cA^	-0.982 (0.872) ^aA^
**Normal** ** saline**	-0.205 (1.162) ^bA^	3.387 (2.051) ^bB^

In this study HCl and H_2_CO_3_ were effective in significantly reducing the surface hardness of MTA on both days 1 and 21. These results are consistent with previous studies [25, 26] and reflect the inverse effect of an acidic environment on the hydration process of this cement. Interestingly, both acids were more effective on MTA after complete setting (day 21). This finding is similar with the results of Nandini *et al.* [22] who showed that H_2_CO_3 _was more effective on reducing the surface hardness of MTA on day 21 compared to day 1.

For CEM cement samples, only HCl and only in day 1 caused a reduction in the microhardness values. Bolhari *et al.* [20] showed that the surface microhardness of CEM cement was reduced significantly by exposure to butyric acid. As in the present study H_2_CO_3 _did not reduce the microhardness of CEM cement, it seems that this cement interacts differently with different acids. 

Although measurement of the microhardness formed the basis of the present investigation, in an attempt to evaluate the effect of NaOCl and HCl on MTA and CEM cement microstructures, a SEM evaluation was also carried out. According to SEM results, although it was not possible to grade the degree of porosity precisely and objectively, exposure to HCl, caused a greater degree of porosity in MTA compared to CEM cement. This is in accordance with the findings of Lee *et al.* [25] and Namzikhah *et al.* [26] who demonstrated that exposure to low pH values produced a greater degree of porosity in MTA.

There were no distinct morphological differences between the groups tested with NaOCl in terms of the internal microstructure. To that end, it should be mentioned that some solvents has been shown to reduce the microhardness of dentin [17]. Therefore, during retrieval of endodontic cements, cautious use of solvents is mandatory to prevent significant alterations in the mechanical and physical properties of the tooth.

## Conclusion

Under the limitation of this study it can be concluded that the solvents were more effective on MTA than on CEM cement. On day 1, all solvent and on day 21, HCl and H_2_CO_3_ reduced the microhardness of MTA. CEM cement was only affected by HCL and NaOCl after 1 day.
